# Supplementary material for: Revealing the Real Role of Etching during Controlled Assembly of Nanocrystals Applied to Electrochemical Reduction of CO2
Source: Nanomaterials (Basel). 2022 Jul 24;12(15):2546. doi: 10.3390/nano12152546 (PMC9332456; doi:10.3390/nano12152546)
Supplement: Supplementary file 1 [file nanomaterials-12-02546-s001.zip › nanomaterials-1715503-supplementary.pdf]

Supporting Information

Revealing the real role of etching during controlled assembly of nanocrystals applied to electrochemical reduction of CO_2

Experimental

Synthesis

Ferric trichloride hexahydrate ($FeCl_3 \cdot 6H_2O$, 99.0 wt.%) and n-hexane (97.0 vol.%) were obtained from Sinopharm Chemical Reagent Co (China, ShangHai). Sodium oleate (NaOA, 65–90 wt.%), oleic acid (OA, 90.0%) and octadecene (ODE, 90%) were obtained from Adamas-Beta. Ethanol (99.7 vol.%) was acquired from Tianjin Zhiyuan Chemical Reagent Co (China, TianJin). Reduced graphene oxide (rGO) is from the Shanxi Institute of Coal Chemistry, Chinese Academy of Sciences (China, ShanXi). All reagents were used directly for further processing.

Firstly, iron oleate was prepared as previously reported [34]. Amounts of 5.4 g $FeCl_3 \cdot 6H_2O$ and 18.25 g sodium oleate were dissolved in a three-necked flask containing 40mL ethanol, 30 mL ultrapure water and 70 mL hexane, heated to 70 °C and held for 4h. After being cooled to room temperature, the iron oleate precursor was obtained by adding 30 mL H_2O in a separatory funnel and washing three times. An amount of 9 g of the above prepared ferric oleate was taken, and 1.425 g oleic acid and 50 g ODA were added to the mixture at 3.3 ℃/min to 120 ℃, evacuated for 30min, then, under Ar flow, heated up to 320 ℃, keep for 30min. The solution was cooled and ethanol was added; it was then centrifuged to obtain the triiron tetraoxide nanocrystals (Fe_3O_4 NPs).

An amount of 2.01g Fe_3O_4 NPs was dispersed with 0.02g rGO ($m_{Fe_3O_4\ NPs}$: m_{rGO} = 100:1) into hexane, volatilized naturally, and sintered in a tube furnace at 500 °C under Ar atmosphere for 2h to obtain Fe_3O_4 nanocrystals loaded to rGO (Fe_3O_4/ rGO-100). The etched Fe_3O_4 NPs/rGO (Fe_3O_4/rGO-H-100) was obtained by etching

and soaking with strong acid (HCl, 1mol L^{-1}) for 12 h to obtain some iron atoms, filtering under reduced pressure, washing 2–3 times using ultrapure water and drying under vacuum at 80 °C for 12h. Dicyandiamide was added and sintered in a tube furnace at 800 °C for 2 h under Ar atmosphere to obtain strongly acid etched nitrogen-doped iron carbide loaded with rGO (N-Fe_3C/rGO-H-100).

Moreover, the comparison samples with different mass ratios of Fe_3O_4 NPs to rGO are similar to the preparation process of N-Fe_3C/rGO-H-100. The comparison samples were synthesized using Fe_3O_4 NPs, Fe_3O_4 NPs to rGO mass ratios of 80 and 100, respectively, and were named as N-Fe_3C, N-Fe_3C/rGO-H-80 and N-Fe_3C/rGO-H-120, respectively.

In addition, the mass ratio of Fe_3O_4 NPs to rGO ($m_{Fe3O4\ NPs}$: m_{rGO}=100:1) was kept constant and the nitrogen doping temperatures (700 and 900°C) were changed to obtain N-Fe_3C/rGO-H-700 and N-Fe_3C/rGO-H-900.

Physical Characterization

Scanning electron microscope (SEM) images were obtained by Hitachi, S4800(Japan, Tokyo). X-ray diffraction patterns were obtained by Rigaku (Showa, Tokyo), Japan for analysis. Transmission electron microscopy (TEM), high resolution TEM and mapping were tested by Tecnai G2 F30 (USA, OR). X-ray photoelectron spectroscopy (XPS) energy spectrum was performed by Thermo Fisher Scientific ESCALAB 250Xi (USA, MA), monochromatic Al Ka (hv =1486.6 eV), power 150W , 500μm beam spot.

Electrochemical CO_2RR Test

An amount of 5mg of catalyst was dispersed in 1mL of 0.25% Nafion solution (V_{Nafion}:V_{H2O}:V_{EtOH} = 1:9:10) and sonicated for 30min. A volume of 70mL of slurry was applied to 1cm × 1cm carbon paper (Shanghai Hesen Reagent, China, ShangHai) and dried naturally as working electrode. The CO_2RR performance test was performed using an H-shaped electrochemical cell with two chambers of 25 mL each, separated by a cation exchange membrane (Nafion 117), using a three-electrode system with a carbon paper coated catalyst as the working electrode, an Ag/AgCl electrode as the reference electrode and a 10 mm × 10 mm platinum mesh electrode as

the counter electrode. The working and reference electrodes were placed at the cathode end of the reaction cell and the counter electrode was placed at the anode end of the reaction cell. When mounting the electrodes, the carbon paper coated with catalyst was placed opposite the counter electrode and the three electrodes were kept at the same height.

The CHI 760E electrochemical workstation was used to perform electrocatalytic reduction of CO_2 at 0.5 M $KHCO_3$. The experiments were performed in a gas-tight, three-electrode system separated by a proton exchange membrane. A volume of 10 mL of a 0.5 M $KHCO_3$ electrolyte was placed in each chamber providing a certain headspace. Before the electrochemical tests, the electrolyte was saturated with CO_2 for 30 min; the CO_2 flow rate was 20 mL/min. The gas phase reduction products were detected at regular intervals using a gas chromatograph (GC Agilent 7890B, USA, Calif.) equipped with a flame ionization detector and a hydrogen flame detector. In this paper, no IR correction was performed during the electrochemical testing. All potentials were converted to reversible hydrogen electrodes relative to each other. The formula for calculating the Faraday efficiency (FE) for the reduced CO_2 product is as follows:

$$FE = \frac{nFC_i vP}{jRT} \quad (S1)$$

where n is the number of transferred electrons, F = 96,500 C mol^{-1}, C_i is the concentration of the target reduction products, ν is the CO_2 gas flow rate, P is the pressure of the injection loop of the column, j is the current density, R = 8.314 J mol^{-1} K^{-1} and T = 273.15 K.

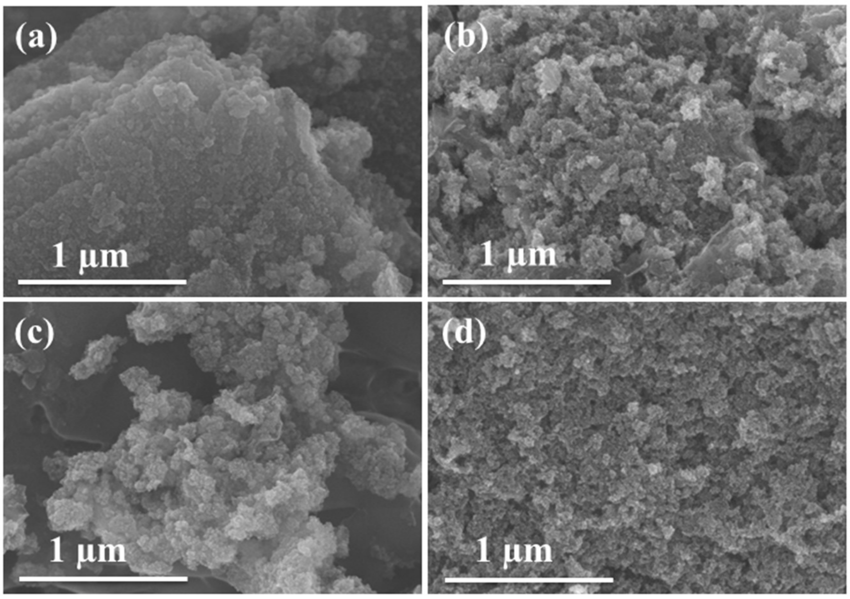

Figure S1. SEM images of (a) Fe_3O_4/rGO-80, (b) Fe_3O_4/rGO-100, (c) Fe_3O_4/rGO-120 and (d) Fe_3O_4.

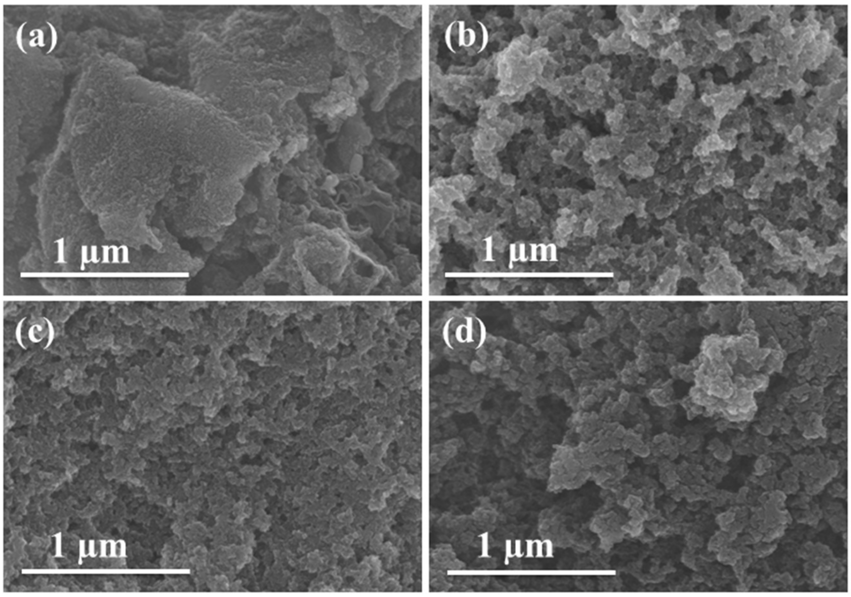

Figure S2. SEM images of (a) Fe_3O_4/rGO-H-80, (b) Fe_3O_4/rGO-H-100, (c) Fe_3O_4/rGO-H-120 and (d) Fe_3O_4-H.

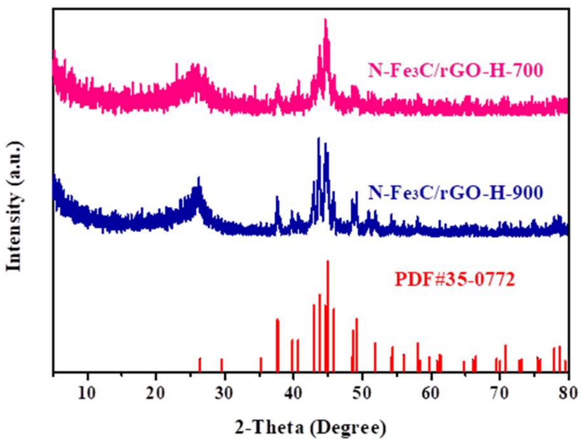

Figure S3. XRD patterns of N-Fe_3C/rGO-H.

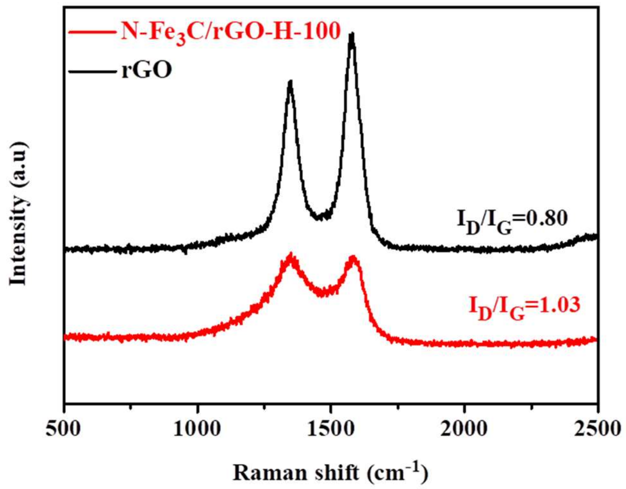

Figure S4. Raman spectra of N-Fe_3C/rGO-H-100 and rGO.

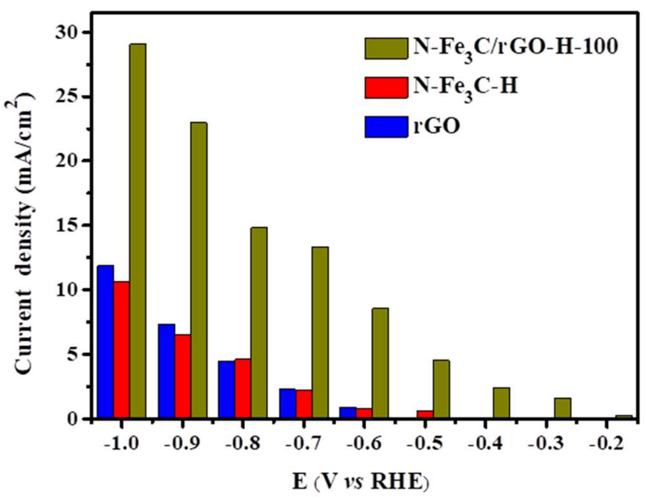

Figure S5. Total current density of N-Fe_3C-H, rGO and N-Fe_3C/rGO-H-100 in CO_2-saturated 0.5 M $KHCO_3$ solution.

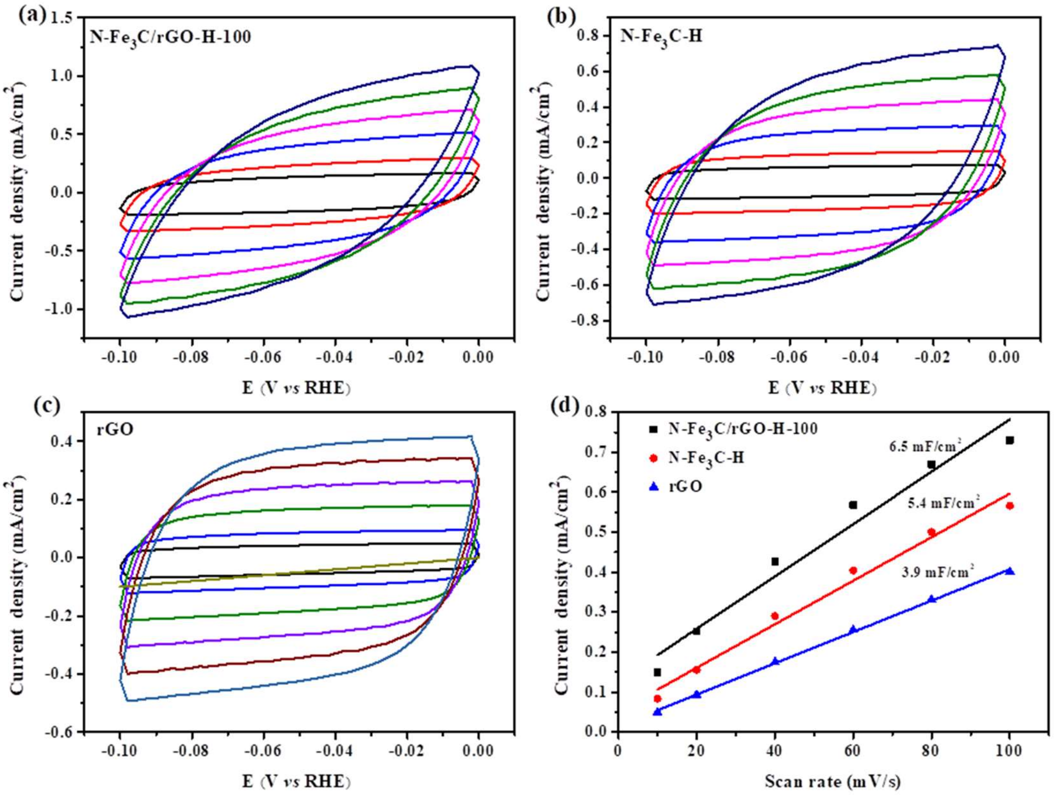

Figure S6. (a-c) Double-layer capacitance of N-Fe_3C/rGO-H-100, N-Fe_3C-H and rGO; (d) determination of double-layer capacitance over a range of scan rates.

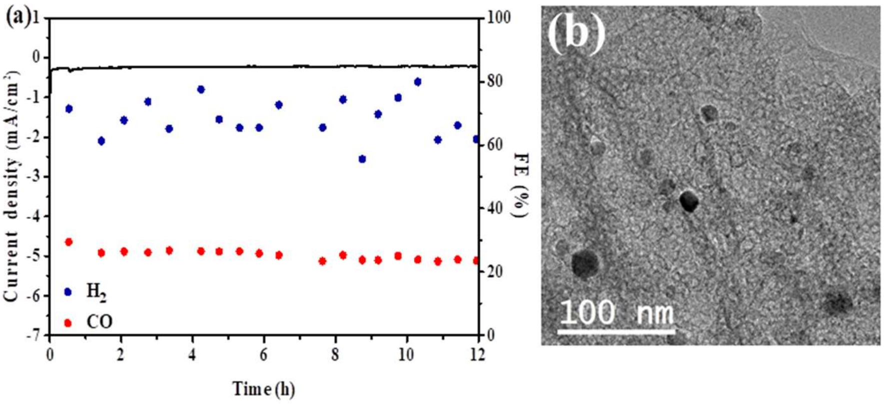

Figure S7. (a) Stability of N-Fe_3C/rGO-H-100 catalyst at −0.4V; (b) TEM image after testing stability.

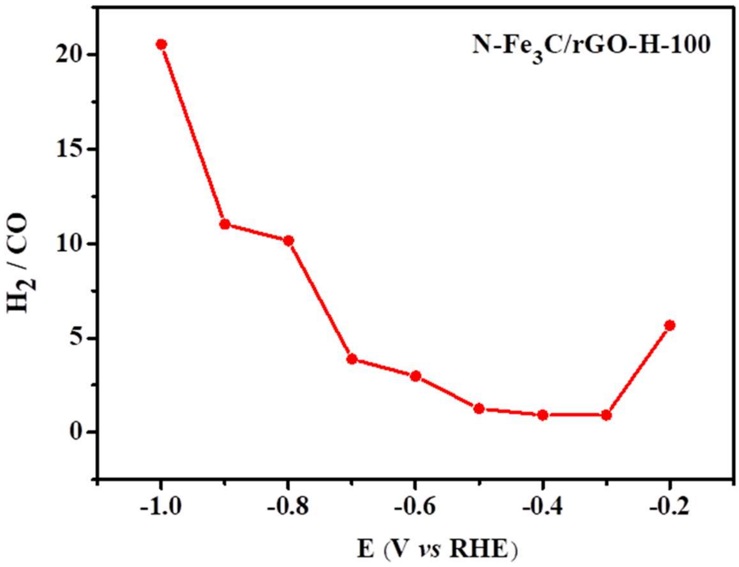

Figure S8. N-Fe_3C/rGO-H-100 syngas components at different applied potentials.

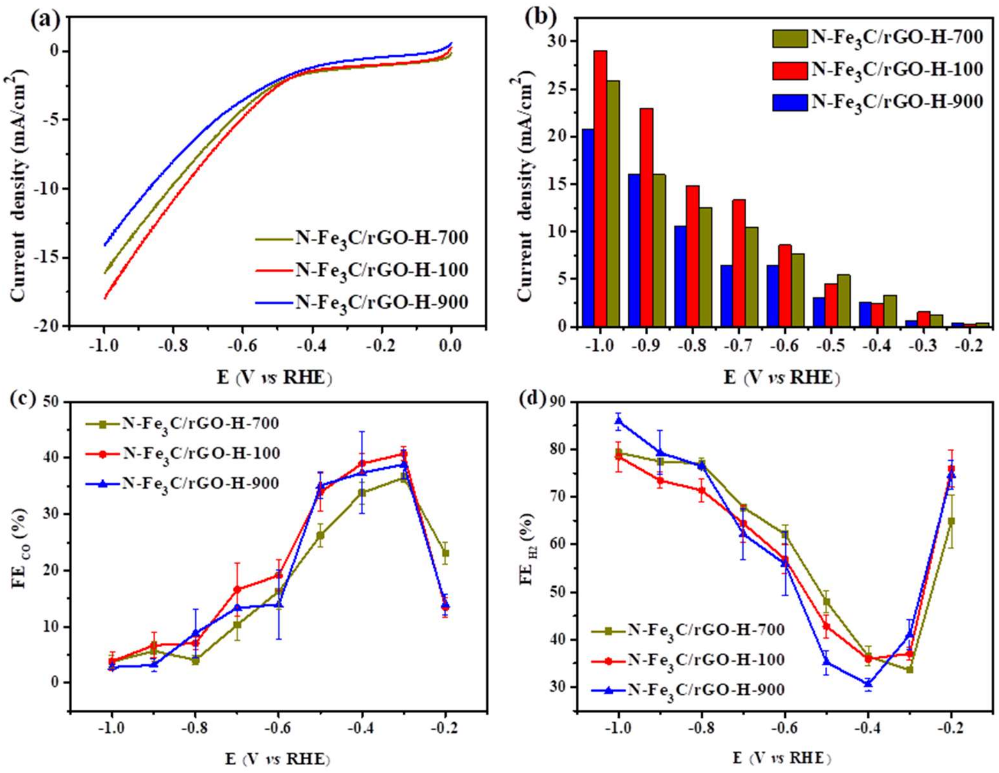

Figure S9. CO_2RR of N-Fe_3C/rGO-H-100, N-Fe_3C-H and rGO: (a) LSV; (b) total current density; (c,d) Faradaic efficiency of CO and H_2 at different nitrogen doping temperatures (700 ~ 900 °C).

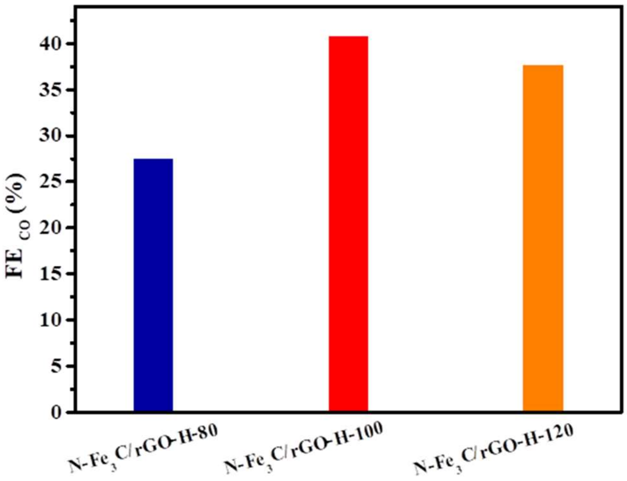

Figure S10. FE_{CO} obtained for N-Fe_3C/rGO-H at –0.3V.

Table S1 The performance comparison of N-Fe_3C/rGO-H-100 and other catalysts.

Catalysts	Potential (V *vs.* RHE)	Ratio of H_2:CO	Electrolytes	Ref.
N-Fe_3C/rGO-H-100	−0.2 ~ −1.0	1 ~ 20	0.5 M $KHCO_3$	This work
PNC	−0.6 ~ −1.0	0.24 ~ 5.25	0.1 M $KHCO_3$	[54]
Ag NWs/carbon sheet	−0.6 ~ −1.8	1.7 ~ 2.15	0.2 M $KHCO_3$	[55]
Co@CoNC-900	−0.5 ~ −0.8	1 ~ 1.5	0.1 M $KHCO_3$	[56]
Cu	−0.6 ~ −1.1	32 ~ 0.56	0.1 M $KHCO_3$	[57]
Ni-hG/Fe-hG	−0.6 ~ −1.1	1~10	0.1 M $KHCO_3$	[58]
Cu/In_2O_3	−0.4 ~ −0.9	4 ~ 0.4	0.5 M $KHCO_3$	[59]
Ni@N–C	−0.57~ −1.07	9 ~ 0.11	0.5 M $KHCO_3$	[60]
CoPc	−0.3 ~ −1.0	0.11	0.5 M $NaHCO_3$	[61]
ZnO/CuO	−0.6 ~ −1.4	4 ~ 1.35	0.5 M $KHCO_3$	[62]
Fe-N-C	−0.3 ~ −1.0	4 ~ 0.33	0.1 M $KHCO_3$	[26]
